# Risk Factors, Health Consequences, and Professional Work of Problematic Mobile Phone Use Among Nurses: A Systematic Review

**DOI:** 10.1155/jonm/3543130

**Published:** 2026-03-24

**Authors:** Gema López-Gutiérrez, Vanesa Gutiérrez-Puertas, Blanca Gómez-Guerrero, Hélder Jaime Fernandes, Stefanos Mantzoukas, Lorena Gutiérrez-Puertas

**Affiliations:** ^1^ Department of Hematology, Torrecardenas University Hospital, Almeria, Andalusia, Spain; ^2^ Department of Nursing, Physiotherapy and Medicine, Faculty of Health Science, University of Almeria, Almeria, Andalusia, Spain, ual.es; ^3^ Research Group PAIDI-TIC 019 “Electronic Communications and Telemedicine”, University of Almeria, Almeria, Andalusia, Spain, ual.es; ^4^ Research Centre for Active Living and Wellbeing (LiveWell), Polytechnic Institute of Bragança, Bragança, Portugal, ipb.pt; ^5^ Department of Nursing, School of Health Sciences, University of Ioannina, Ioannina, Greece, uoi.gr; ^6^ Research Group PAIDI-HUM 061 “Experimental and Applied Neuropsychology”, University of Almeria, Almeria, Andalusia, Spain, ual.es

**Keywords:** clinical setting, nomophobia, nurse, problematic mobile phone use, smartphone addiction

## Abstract

**Background:**

Problematic mobile phone use is an emerging public health issue, the prevalence of which has increased among nurses.

**Aim:**

To synthesize and describe knowledge on problematic mobile phone use by nurses, its consequences, and strategies for addressing this phenomenon.

**Design:**

A systematic review was conducted following the checklist Preferred Reporting Items for Systematic Reviews and Meta‐Analysis (PRISMA) statement. This systematic review has been registered in PROSPERO (CRD420251052591).

**Methods:**

Four electronic databases were systematically searched from their inception to September 2025. The article′s reference lists were also manually searched. The study selection was carried out in three stages, with two reviewers independently analyzing the data and resolving disagreements. The quality assessment utilized the Mixed Method Appraisal Tool, considering the criteria established for each study design.

**Results:**

Sixteen studies from four online databases were selected, the majority of which were cross‐sectional and descriptive. The risk factors for problematic mobile phone use, the negative consequences for mental and physical health, and the clinical work of nurses were highlighted, such as a combination of strategies to prevent and mitigate problematic mobile phone use in the clinical setting.

**Conclusion:**

The problematic mobile phone use of nurses negatively affects their mental and physical health, as well as their performance in the clinical setting.

**Implication for nursing management:**

The findings of this study may inform the need for nursing managers to develop and implement strategies to prevent and mitigate the problematic use of these devices among nurses and ensure the appropriate use of mobile phones in the clinical setting.

## 1. Introduction

Mobile phones have become an indispensable part of people’s lives due to their multiple features, such as size, portability, and access to the internet at any time and place [1]. These devices have changed the way humans interact, as they provide the opportunity to communicate and receive messages, as well as access, store, and share information instantly [2]. These features have contributed to their global expansion, with an estimated 7.1 billion mobile phone users worldwide [3].

Nurses in clinical settings use mobile phones for professional purposes, such as communicating with members of the multidisciplinary team to consult on clinical matters concerning patients [4] or with patients and their families to coordinate care [5]. Another common use is the search for relevant clinical information on the internet or through mobile applications to support clinical decision‐making [6]. Similarly, nurses use these devices for patient care−related issues such as storing information, taking notes, photographing clinical records, and recording appointments [7]. The use of mobile phones for work purposes is positively associated with productivity and quality of care [4]. However, the use of these devices in the clinical setting can cause work distractions, threaten privacy, and interfere with professionalism, compromising patient safety and quality of care [8, 9].

Nurses have increased their use of mobile phones in clinical settings for personal purposes, with the main activities being the use of social media and messaging [7, 10]. A study conducted in Germany revealed that 63% of nurses use mobile phones in clinical settings exclusively for personal purposes [11]. Mobile phone use leads to tolerance, increasing the need for more time spent on the phone and causing addictive behaviors such as problematic mobile phone use [2]. Mobile phone addiction is described as “problematic mobile phone use,” also referred to as “smartphone addiction,” “excessive mobile phone use,” “mobile phone dependence,” or “nomophobia” [12]. Problematic mobile phone use is considered an emerging public health concern [13]. This behavior regarding mobile phones is characterized by excessive or uncontrolled use, anxiety when unable to use the device, fear of unavailability, dependence to relieve negative emotions, and interference with interpersonal relationships or work activities, leading to symptoms similar to those of an addiction [14]. Nomophobia is the uncontrollable fear of leaving home without a mobile phone or losing connection, causing anxiety and worry [15]. A recent review reveals that the total prevalence of nomophobia among nurses is 68.15% [16]. Problematic mobile phone use can affect mental and physical health, triggering sleep and behavioral disorders, depression, neck pain, and fatigue, thus negatively impacting nurses’ work performance [17, 18].

In healthcare settings, nurses are essential to ensuring optimal patient care. The increased use of mobile phones among nurses during their working hours reduces the time dedicated to clinical tasks and increases interruptions and distractions during their clinical work, which can negatively affect efficiency, privacy, and patient safety [4, 8]. Nurses’ dependence on mobile phones can lead to the development of addictive behaviors such as problematic use, mobile phone addiction, or nomophobia, which could interfere with their health and clinical work [10]. To our knowledge, no studies have been conducted that summarized the available evidence on the consequences of problematic mobile phone use among nurses. Several studies have associated problematic mobile phone use with constructs related to physical and mental well‐being, as well as aspects related to nurses’ professional performance [7, 9]. Therefore, understanding the consequences of problematic mobile phone use among nurses is crucial for designing and implementing effective strategies to address these adverse outcomes and reduce their severity. In this context, a systematic review focusing on nurses provides a structured summary of (i) risk factors, (ii) consequences for physical and mental health, (iii) repercussions on their clinical performance, and (iv) strategies to reduce its impact, which could be a useful starting point for guiding interventions to prevent or mitigate problematic mobile phone use, as well as for developing organizational policies on mobile phone use in the clinical setting. Thus, the aim of this systematic review was to synthesize and describe evidence on problematic mobile phone use among nurses, specifically the risk factors associated with problematic mobile phone use, the negative consequences, and to identify available strategies to address it in the clinical setting.

## 2. Methods

### 2.1. Design

A systematic review was conducted, including studies up to September 30, 2025 (date of the last search). The checklist Preferred Reporting Items for Systematic Reviews and Meta‐Analysis (PRISMA) statement was followed to report this systematic review [19]. The review protocol was registered in the International Register of Systematic Reviews (PROSPERO ID: CRD420251052591). Primary studies were reviewed that addressed the following research questions: (a) What are the risk factors among nurses with problematic mobile phone use? (b) What are the negative consequences of problematic mobile phone use among nurses? (c) What strategies are available to address this issue in clinical settings?

### 2.2. Search Strategy

The systematic review search was guided by population, exposure, and outcomes (PEOs) criteria, where *P* = nurse, *E* = problematic mobile phone use, and *O* = consequences/effects [20]. Systematic searches were conducted to identify relevant studies between January and September 2025 in four electronic databases: PubMed, Cumulative Index of Nursing and Allied Health Literature (CINAHL), Scopus, and Web of Science. The final search was performed in September 2025, and studies published up to this date were eligible for inclusion. Specific keywords and terms were used in the searches, MeSH terms and accessible terms, combined with Boolean operators “AND” and “OR” (Table 1).

**TABLE 1:** Descriptor combinations in each database used.

Database	PubMed	Scopus	Web of Science	CINAHL
Nurse	(((nurse [Title/Abstract]) OR (administrator, nurse [MeSH Terms])) OR (nurs^∗^[Title/Abstract])	TITLE‐ABS‐KEY (nurses) OR TITLE‐ABS‐KEY (nurs^∗^) OR TITLE‐ABS‐KEY (administrator AND nurse)	TS= (nurse OR administrator nurse OR nurs^∗^))	(nurses OR administrator nurse OR nurs^∗^)
Mobile phone problematic use	AND (((((((((((((smartphone[MeSH Terms]) OR (cellular phone[MeSH Terms])) OR (nomophobia[Title/Abstract])) OR (problematic mobile phone use[Title/Abstract])) OR (problematic smartphone use[Title/Abstract])) OR (problematic cellular phone use[Title/Abstract])) OR (smartphone addiction[Title/Abstract])) OR (cellular phone addiction[Title/Abstract])) OR (mobile phone addiction[Title/Abstract])) OR (smartphone excessive use[Title/Abstract])) OR (Cellular phone Excessive use[Title/Abstract])) OR (mobile phone Excessive use[Title/Abstract]) AND (fft[Filter]))	AND TITLE‐ABS‐KEY (problematic AND mobile AND phone) OR TITLE‐ABS‐KEY (problematic AND cellular AND phone AND use) OR TITLE‐ABS‐KEY (cellular AND phone AND addiction) OR TITLE‐ABS‐KEY (mobile AND phone AND addiction) OR TITLE‐ABS‐KEY (cellular AND phone AND excessive AND use) OR TITLE‐ABS‐KEY (mobile AND phone AND excessive AND use)	AND ((TS = (Nomophobia OR problematic mobile phone use OR problematic smartphone use OR problematic cellular phone use OR Smartphone addiction OR Cellular phone addiction OR Mobile phone addiction OR Smartphone Excessive use OR Cellular phone Excessive use OR mobile phone Excessive use))	AND (Nomophobia OR problematic mobile phone use OR problematic smartphone use OR problematic cellular phone use OR Smartphone addiction OR Cellular phone addiction OR Mobile phone addiction OR Smartphone Excessive use OR Cellular phone Excessive use OR mobile phone Excessive use)
Clinical setting	AND ((clinical setting [Title/Abstract]) OR (hospital [Title/Abstract]) AND (fft[Filter]))	AND TITLE‐ABS‐KEY (clinical AND setting) OR TITLE‐ABS‐KEY (hospital)	AND TS = (hospital OR clinical setting)	AND (hospital or clinical setting)
Results number	197	22	70	12

^∗^Truncation symbol (wildcard) used to capture multiple word endings and spelling variants.

### 2.3. Selection of Studies and Eligibility Criteria

The inclusion criteria established were as follows: (a) primary research studies; (b) published in English, Spanish, Portuguese, or Greek; (c) from the inception date of the databases consulted up to September 2025; (d) study participants were nurses; (e) studies addressing addiction, problematic or excessive mobile phone use, or nomophobia; and (f) studies evaluating risk factors, negative consequences, and strategies to address this issue. The exclusion criteria were as follows: (a) studies involving nursing students, other healthcare professionals, or mixed samples of healthcare workers; (b) studies focused on the benefits of mobile phone use in clinical settings; (c) systematic reviews, dissertations, discursive papers, editorials, conference papers, case reports, study protocols, reports, and chapters of books. Potential additional studies were identified by reviewing the reference lists of all studies.

### 2.4. Data Screening

The studies retrieved from the database search were selected in three stages: by title, abstract, and full‐text. The eligibility process was carried out independently by two authors (GLG and BGG) and in duplicate. In case of discrepancy, a third author (LGP) was consulted to reach a consensus based on the aim of the study and research question.

### 2.5. Quality Appraisal

All eligible studies were critically read and assessed for methodological quality using Mixed Methods Appraisal Tool (MMAT) [21], which is a reliable and widely used tool for evaluating different types of research designs, such as mixed‐methods, randomized, nonrandomized, quantitative descriptive, and qualitative studies. The studies were assessed with MMAT, selecting the appropriate category for appraisal and then rating the criteria by rating “Yes,” “No,” or “Cannot tell.” Overall, the methodological quality of the included studies was considered moderate to good, with a range from 60% to 100% according to the MMAT classification [21]. Two researchers independently assessed the quality of the selected studies, and a third researcher was consulted to discuss any discrepancies until a consensus was reached.

### 2.6. Data Extraction and Synthesis

Data from the included studies were extracted and tabulated by three authors independently according to: (a) author; (b) year of publication; (c) country; (d) aims; (e) study design; (f) sample size, specifying the percentage of women in the study; (*g*) age in years of the study sample (mean, standard deviation, or range); (h) instruments for assessing problematic use, addiction, and excessive mobile phone use or nomophobia; (i) main findings (including prevalence of problematic use, addiction, excessive mobile phone use or nomophobia, mean score and standard deviation of problematic use, hours of mobile phone use per day, variables related to excessive mobile phone use, negative consequences of problematic mobile phone use, and strategies to address the issue); and (*j*) methodological quality. Discrepancies were resolved through discussion until consensus was reached, consulting a fourth researcher when that persisted. Due to the heterogeneity of the studies selected for analysis and synthesis, descriptive and narrative analyses were performed to synthesize the extracted data, in accordance with the aim.

## 3. Results

### 3.1. Selection of Studies Included in the Systematic Review

**FIGURE 1:**
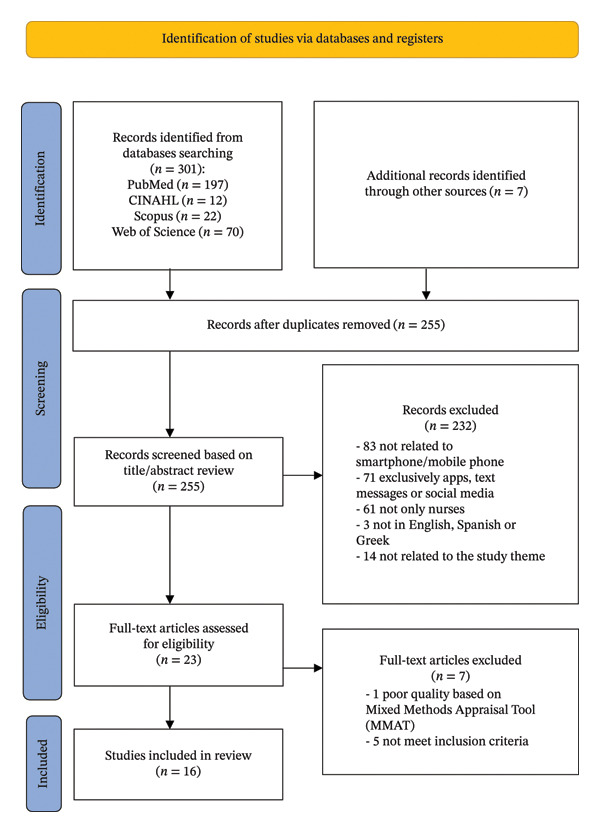
PRISMA 2020 flow diagram for new systematic reviews, which included searches of databases and registers of the review process.

First, a search was conducted in four online databases, identifying 301 studies (PubMed [*n* = 197], CINAHL [*n* = 12], Scopus [*n* = 22], Web of Science [*n* = 70]). Seven studies were identified through a review of reference lists during the initial search. After initial assessment, fifty‐three studies were excluded due to duplication. Subsequently, studies were screened by title and abstract, excluding 232 studies. Twenty‐three studies were selected for full‐text reading to determine their eligibility. Seven studies were excluded for not specifically focusing on problematic use, addiction, excessive mobile phone use, or nomophobia, and one for not meeting the MMAT quality criteria. Only sixteen studies met the inclusion criteria and were included in this review. The selection process is illustrated in Figure 1.

### 3.2. Study Characteristics

The main characteristics of the studies included in this review are presented in Table 2. The studies included in the systematic review were published between 2015 and 2025. Of the 16 studies included, fifteen (94.44%) of these studies were quantitative studies, among which all of them were cross‐sectional observational, and one (5.56%) was a qualitative study. Among the 16 studies included, three were conducted in Western countries, including the United States (*n* = 1, 6.66%) and Italy (*n* = 2, 13.33%); seven in Eastern countries, China (*n* = 4, 26.66%), Philippines (*n* = 1, 6.66%), India (*n* = 1, 6.66%), and Iran (*n* = 1, 6.66%); and six in Middle East countries, Turkey (*n* = 5, 33.32%) and Egypt (*n* = 1, 6.66%). Fifteen studies used validated questionnaires to analyze the characteristics of the variables studied, and one study used a semistructured interview. Regarding problematic mobile phone use, most of the research assessed nomophobia (*n* = 8), followed by mobile phone addiction (*n* = 4) and problematic mobile phone use (*n* = 4). The sample sizes of the included studies ranged from 23 [36] to 1418 nurses [34]. The gender represented most was female, with a percentage that ranged from 32.6% [31] to 100% [30]. The nurses considered for data collection ranged in age from 18 [22] to 69 years [23].

**TABLE 2:** Summary of characteristics of the included studies.

Authors, years, and country	Aims	D	Sample size/participants (gender, age)	Data collection tools/Type problematic mobile phone	Main results	Statistical results (risk factors and outcomes)	MMAT score
Bülbüloğlu et al. [22], Turkey	To identify nomophobic behaviors of nurses working in surgical clinics and to investigate the effects of these behaviors on time management and psychological well‐being	C	*N* = 314. 65% were women. The average age of participants was 32.47 (SD = 5.59) Age range: 18–47 years old. 41% of respondents were between 30 and 35 years old	‐ Nomophobia Scale (Yildirim and Correia, 2015) ‐ Free Time Management Scale (Akgül and Karaküçük, 2015) ‐ Brief Resilience Scale (Dögan, 2015)	Older participants tended to report lower levels of nomophobia. In addition, mobile phone use at work significantly increased nomophobia, decreased the level of psychological well‐being and showed a negative effect on time management. Nomophobia also affected nurses’ psychological well‐being by increasing their fear of not being able to access information, giving up comfort, and not being able to communicate	Risk factors ⟶ nomophobia: Age: β = −0.115^∗^ (−). Daily smartphone use: β = 0.355^∗∗∗^ (+). Use while on duty: β = −0.164^∗∗^ (−). Educational level: β = 0.112^∗^ (+). Nomophobia ⟶ mental health: Psychological well‐being: *R* = −0.281^∗∗∗^, *R*2 = 0.133 (−). Unable to communicate subscale ⟶ well‐being: β = −0.232^∗^ (−). Unable to access information subscale ⟶ well‐being: β = 0.194^∗^ (+). Nomophobia ⟶ time management: Use while on duty ⟶ time management: β = −0.163^∗∗^ (−)	100% (5/5) High quality

Conant et al. [23], USA	To describe how nurses use mobile phones while providing direct patient care and to identify generational differences	C	*N* = 335. 89.6% were women. The average age of participants was 41.77 (SD = 12.95) Age range: 18–69 years old. 38.5% of participants were between 36 and 50 years old; 31.6% were from 18 to 35 years	An ad hoc questionnaire was developed and validated. It had 11 items about frequency of use of mobile phone, its impact on work and perceptions of use	In general, participants considered that mobile phone were not a major distraction during work. Even so, there were generational differences related to the presence of these devices in patient care. Older nurses perceived mobile phone use in the clinical setting more problematic	Risk factors ⟶ problematic use: Age/generation: millennials 60.4% vs. Gen X 43.4% vs Boomers 22.3% no distraction (−)a. Problematic use ⟶ clinical practice: Self‐distraction: millennials 87.8%, Gen X 73.5%, boomers 52.2% no distraction (−)a. Work performance: millennials 86.8%, Gen X 75.9%, boomers 58.5% no impact (−)a Problematic use ⟶ patient care: Negative effect: boomers 74.5% perceive negative effect vs. millennials 68.9% no effecta.	60% (3/5) Moderate–low quality

Di Simone et al. [24], Italy	To explore nurses’ opinions and the impact of mobile phone use during the work shift and how this may affect care	C	*N* = 778. 73.5% were women. The average age of participants was 35.7 (SD = 9.17) Age range was divided into three groups: under 30 years old (36.5%), between 31 and 40 years (23.1%), and the last one, 41–50 years old (38.6%)	Nurses’ Use of Personal Communication Devices Questionnaire (Di Muzio et al., 2019)	The use of these devices for nonwork‐related activities was more frequent in men, and it negatively affected their work performance. Furthermore, younger nurses reported being more distracted by their use than older ones	Risk factors ⟶ problematic use: Gender (male): males report more negative impact on performance, t = 3.358^∗∗∗^ (+). Age (younger < 30 y): younger nurses more distracted, F (2.759) = 4.902^∗∗^ (−). Work experience (1–10 y): less experience more distracted, F (2.773) = 3.878^∗^ (−). Educational level (nonuniversity):more negative impact on performance, *t* = 1.028^∗^ (+)a. Problematic use ⟶ clinical practice: Distraction: younger nurses (< 30 y) more distracted, F (2.759) = 4.902^∗∗^ (−). Performance improvement belief: younger nurses believe smartphones improve performance, F (2.760) = 12.702^∗∗∗^ (+). Problematic use ⟶ patient safety: Medical errors: 47% agree smartphones increase error probabilitya. Patient safety belief: Older nurses believe less in safety improvement, *F* (2.758) = 7.790^∗∗^ (−)	80% (4/5) Moderate–high quality

Dinçer and Çelik Ínce [25], Turkey	To assess the levels of nomophobia among working nurses and midwives in a gynecology and children’s hospital, as well as to explore the impact of nomophobia on their lives	C	*N* = 112. 98.2% were women. The average age of participants was 34.5 (SD = 9.98) Age range was divided into three groups: under 30 years old (51.8%), between 30 and 45 years (26.8%), and older than 45 years old (21.4%)	Nomophobia Questionnaire (Yildirim and Correia, 2015)	54.5% of the participants reported a moderate level of nomophobia. Age, years of experience and habits of checking mobile phone upon waking up influenced the level of nomophobia	Risk factors ⟶ nomophobia: Age (< 30 y): higher nomophobia vs. > 45 y, *F* = 3.698^∗^ (−). Work experience (≤ 10 y): higher nomophobia vs. > 20 y, *F* = 3.047^∗^ (−). Education: ns Checking phone immediately after waking total score *F* = 3.876^∗^ (+). Checking before sleep: “sacrificing comfort” subdimension, *F* = 6.259^∗∗^ (+). Phone use habit before sleep ⟶ higher discomfort, *F* = 1.553 (n/s). Work type (night shifts) ⟶ higher total score, *F* = 2.091^∗^ (+). Nomophobia prevalence: Mean ± SD = 68.45 ± 24.62 (moderate level), 54.5% moderate, 9.8% high. Nomophobia ⟶ health outcomes: Not directly tested; authors conclude impact on mental and physical health, especially among younger and less experienced nursesa	100% (5/5) High quality

El‐Sayed et al. [26], Egypt	To assess the prevalence of cell phone addiction and procrastination behavior among nurses	C	*N* = 360. 63.1% were women. The average age of participants was 28.29 (SD = 7.34) Age range: 20–59 years old	‐ Smartphone Addiction Inventory (Lin et al., 2014) ‐ The New Active Procrastination Scales (Choi and Moran, 2009) ‐ The Unintentional Procrastination Scale (Fernie et al., 2017)	55% of nurses indicated moderate perceived mobile phone addiction, 80% active procrastination behaviors and 45.3% passive procrastination. A significant positive statistical difference was found between the level of mobile phone addiction and procrastination of the nurses	Risk factors ⟶ smartphone addiction: Smartphone addiction ↔ active procrastination: *r* = 0.533^∗∗∗^ (+). Work experience (shorter): associated with higher procrastination, *β* = −0.468^∗∗^ (−). Smartphone addiction prevalence: mean ± SD = 66.5 ± 16.3 (moderate level). Smartphone addiction ⟶ clinical practice (procrastination): Active procrastination: *r* = 0.533^∗∗∗^ (+). Passive procrastination: *r* = 0.468^∗∗∗^ (+). Smartphone addiction explained *R*2 = 0.319 (25% variance) in active procrastination *β* = 0.516^∗∗∗^ (+). Smartphone addiction explained *R*2 = 0.270 (18% variance) in passive procrastination *β* = 0.437^∗∗∗^ (+). Correlation between active and passive procrastination: *r* = −0.582^∗∗∗^ (−)	100% (5/5) High quality

Hoşgör et al. [27], Turkey	To investigate the relationship between nomophobia, FoMO and perceived work overload in nurses in Turkey.	C	*N* = 178. 90% were women. The average age of participants was 30.5 (SD = 7.30) Age range was divided into three groups: the first one 20–29 years old (59%), the second 30–39 years old (24.2%), and the last one, 40 years old or older (16.9%)	‐ Nomophobia Questionnaire (Yildirim & Correia, 2015) ‐ Fear of Missing Out Scale (Gökler et al., 2016) ‐ Perceived Work Overload Scale (Erdem et al., 2017)	Participants had a moderate level of nomophobia (37.6%). It was observed that, as the level of nomophobia increased, the FoMO levels and work overload perceived increased. In addition, nurses’ levels of FoMO and nomophobia were found to decrease with increasing age and work experience	Risk factors ⟶ nomophobia/FoMO: Age ↔ nomophobia: *r* = −0.238^∗∗^ (−). Experience ↔ nomophobia: *r* = −0.254^∗∗^ (−). Age ↔ FoMO: *r* = −0.232^∗∗^ (−). Experience ↔ FoMO: *r* = −0.299^∗∗∗^ (−). Nomophobia/FoMO prevalence: mean ± SD Nomophobia = 50.8 ± 17.26 (moderate level). Mean ± SD FoMO = 19.5 ± 7.05 (moderate). Nomophobia ⟶ clinical practice (perceived work overload): nomophobia ⟶ FoMO: *r* = 0.558^∗∗^ (+). Nomophobia ⟶ perceived work overload: *r* = 0.270^∗∗^ (+). FoMO ⟶ perceived work overload: *r* = 0.154^∗^ (+). Regression model (predictors of perceived work overload): Nomophobia *β* = 0.267^∗∗^ (+). FoMO *β* = 0.005 (ns) Model *R*2 = 0.062 (6% variance explained)	(3/5) 60% Moderate–low quality

Kapikiran et al. [28], Turkey	To analyze the effects of emergency nurses’ nomophobic behaviors on their perceptions of clinical decision‐making	C	*N* = 186. 79.6% were women. The average age of participants was 33.37 (SD = 7.15) Age range: 21–53 years old	‐ Nomophobia Questionnaire (Yildirim and Correia, 2015) ‐ Clinical Decision‐Making in Nursing Scale (Durmaz‐Edeer and Sarikaya, 2015)	Emergency nurses were found to have moderate levels of nomophobia. Furthermore, it was observed that the levels of perceived clinical decision‐making decreased as the levels of nomophobia increased	Risk factors ⟶ nomophobia: Daily smartphone use duration ⟶ nomophobia: *r* = 0.253^∗∗∗^ (+). Gender (female/male): ns. Age ⟶ nomophobia: *r* = −0.082, ns. Work experience ⟶ nomophobia: *r* = −0.111, ns. Nomophobia ⟶ clinical practice (clinical decision‐making): Nomophobia ⟶ clinical decision‐making: *r* = −0.730^∗∗∗^(−). Regression model: *β* = −0.730^∗∗∗^ (−). *R*2 = 0.530 (nomophobia explains 53% of variance in decision‐making perceptions). F(1.184) = 209.330^∗∗∗^. Nomophobia subscales ⟶ Clinical decision‐making: “Not being able to communicate” ⟶ strong negative correlation, *r* = −0.714^∗∗^ (−). “Giving up convenience” ⟶ *r* = −0.714^∗∗^ (−). “Search for information and assimilation of new info” (decision‐making dimension) ⟶ *r* = −0.754^∗∗^ (−).	100% (5/5) High quality

Ma et al. [18], China	To describe mobile phone addiction, occupational burnout, nursing adverse events, and their relationship in novice Chinese nurses	C	*N* = 366. 95.1% were women. The average age of participants was 23.03 (SD = 1.59) Age range: 20–28 years old	‐ Mobile Phone Addiction Index (Huang et al., 2014) ‐ Maslach Burnout Inventory Human Services Survey (Chen, 2000)	The results showed that the frequency of adverse events was related to high levels of mobile phone addiction, and more than half of the participants had high levels of occupational burnout. Furthermore, nurses with higher levels of mobile phone addiction reported higher levels of burnout	Problematic use ⟶ mental health (burnout): Mobile phone addiction and burnout: *r* = 0.33^∗∗^ (+). Subdimensions with significant positive correlations: Emotional exhaustion: *r* = 0.268–0.331^∗∗^ (+). Depersonalization: *r* = 0.260–0.298^∗∗^ (+). Problematic use ⟶ Clinical practice (adverse events): Nurses with higher mobile addiction reported more frequent nursing adverse events, including: Wrong medication: *t* = −3.62^∗∗∗^ (+). Pressure ulcer: *t* = −3.09^∗∗^ (+). Patients fall with injury: *t* = −3.06^∗∗^ (+). Nosocomial infection: *t* = −2.20^∗^ (+). Unplanned extubation: *t* = −3.58^∗∗∗^ (+). Higher addiction scores among nurses reporting those events. Mobile phone addiction and productivity loss: *r* = 0.331^∗∗^ (+).	100% (5/5) High quality

Ma et al. [29], China	To explore the relationship between mobile phone addiction among newly graduated nurses, burnout and work procrastination	C	*N* = 220. 93.6% were women. The average age of participants was 24.5 (SD = 1.73) Age range: 20–28 years old	‐ Mobile Phone Addiction Index Scale (Huang et al., 2014) ‐ General Procrastination Scale (Lay, 1986) ‐ MBI‐GS Maslach Burnout Inventory General Survey (Li and Shi, 2003)	The level of mobile phone addiction was moderate. As participants’ work procrastination increased, their addiction to mobile phone increased. The addiction to these devices and nurses’ procrastination were predictive of the burnout levels	Risk factors ⟶ burnout (in the sample assessed for mobile phone addiction): Age ⟶ emotional exhaustion: p = 0.003b. Marital status ⟶ emotional exhaustion: p = 0.002b. Cynicism: p = 0.042b Reduced professional efficacy: p = 0.005b. Education level ⟶ emotional exhaustion: nsb. Gender ⟶ burnout: nsb. Mobile phone addiction ⟶ mental health (burnout): Mobile phone addiction ↔ work procrastination: *r* = 0.348^∗∗^ (+). Mobile phone addiction ↔ emotional exhaustion: r = 0.360^∗∗^ (+). Mobile phone addiction ↔ cynicism: *r* = 0.322^∗∗^ (+). Regression model: Mobile phone addiction ⟶ emotional exhaustion *β* = 0.278^∗∗∗^ Model R2 = 0.189 (18.9% variance explained). Mobile phone addiction ⟶ cynicism *β* = 0.428^∗∗∗^ Model R2 = 0.258 (25.8% variance explained). Mobile phone addiction ⟶ clinical practice: Mobile phone addiction ↔ reduced professional efficacy: *r* = −0.136^∗^ (−). Model *R*2 = 0.062 (6% variance explained)	100% (5/5) High quality

Pormehr et al. [30], Iran	To investigate the role of sleep quality as an intermediary in the relationship between mobile device use before sleep and nursing performance	C	*N* = 200. 100% were women. The average age of participants was 33.92 (SD = 7.93) Age range: 22–55 years old	‐ Two questions about the use of mobile phone. ‐ Pittsburgh Sleep Quality Index (Farrahi‐Moghaddam et al., 2012) ‐ Epworth Sleepiness Scale (Sadeghniiat‐Haghighi et al., 2013) ‐ Occupational Cognitive Failure Questionnaire (Hassanzadeh‐Rangi et al., 2012) ‐ Nursing Error Questionnaire (Baghaei et al., 2012)	Participants who used their mobile phone longer (both during the day and before bedtime) reported poor sleep quality, higher levels of daytime sleepiness, more information processing failures at work and more nursing errors. Moreover, it was found that older participants tended to report moderately lower daytime use of mobile phone. However, the use of these devices before sleep did not decrease	Risk factors ⟶ PMDs use: Age ⟶ Daily PMDs use (hour/day): r = −0.27^∗∗∗^ (−). PMDs use ⟶ mental health (sleep): Daily PMDs use ⟶ SQ: r = 0.25^∗∗∗^ (+). Daily PMDs use ⟶ DS: r = 0.22^∗∗∗^ (+). Presleep PMDs use ⟶ SQ: r = 0.38^∗∗∗^ (+). Presleep PMDs use ⟶ DS: r = 0.26^∗∗∗^ (+). SQ ⟶ DS: *r* = 0.54^∗∗∗^ (+) Path model (direct/indirect): Presleep PMDs use ⟶ SQ: β = 0.38^∗∗∗^ (+). Presleep PMDs use ⟶ DS: β = 0.22^∗^ (+). SQ ⟶ DS: β = 0.43^∗∗∗^ (+). Indirect (Presleep PMDs use ⟶ SQ ⟶ DS): β = 0.16^∗∗^ (+). Age ⟶ SQ: β = −0.015 (ns)c. Model R2: SQ R2 = 0.145 (14.5% variance explained); DS R2 = 0.305 (30.5% variance explained). PMDs use ⟶ Clinical practice: Daily PMDs use ⟶ OCF: r = 0.23^∗∗∗^ (+). Daily PMDs use ⟶ NE: r = 0.18^∗∗∗^ (+). Presleep PMDs use ⟶ OCF: r = 0.31^∗∗∗^ (+). Presleep PMDs use ⟶ NE: r = 0.31^∗∗∗^ (+). Path model (direct/indirect): Presleep PMDs use ⟶ OCF: β = 0.29^∗∗^ (+). Presleep PMDs use ⟶ NE: β = 0.43^∗∗∗^ (+). Indirect (Presleep PMDs use ⟶ SQ ⟶ OCF): β = 0.10^∗∗^ (+). Indirect (presleep PMDs use ⟶ SQ ⟶ NE): β = 0.06 (ns)c. Model R2: OCF R2 = 0.216 (21.6% variance explained); NE R2 = 0.253 (25.3% variance explained)	80% (4/5) Moderate–high quality

Sharma et al. [31], India	To assess the level of nomophobia of nurses and its relationship with self‐efficacy and mindfulness	C	*N* = 420. 32.6% were women. The average age of participants was 27.99 (SD = 4.69) Age range: 63.3% of nurses were between 27 and 32 years old, while 31% were from 21 to 26 years old	‐ Nomophobia Questionnaire (Yildirim an Correia, 2015) ‐ Mindfulness Attention Awareness Scale (Brown and Ryan, 2003) ‐ General Self‐efficacy Scale (Schwarzer and Jerusalem, 1995)	99.5% of the participants showed nomophobia. 53.3% reported high levels of self‐efficacy and 52.6% mindfulness. The higher the level of nomophobia, the lower the self‐efficacy and mindfulness of the nurses	Risk factors ⟶ nomophobia: Gender ⟶ nomophobia: p = 0.02^∗^d. Area of placement ⟶ nomophobia: p = 0.005^∗∗^d Duration of smartphone being used ⟶ nomophobia: p = 0.001^∗∗∗^d Daily average time spent on a Smartphone ⟶ nomophobia: p = 0.001^∗∗∗^d. Nomophobia ⟶ mental health (mindfulness): Nomophobia ↔ mindfulness: r = −0.289^∗∗^ (−). Nomophobia ⟶ clinical Practice (self‐efficacy): Nomophobia ↔ self‐efficacy: r = −0.278^∗∗^ (−)	100% (5/5) High quality

Uguz and Bacaksiz [32], Turkey	To define the levels of nomophobia, compare these levels according to sociodemographic data and mobile phone use behaviors, as well as to investigate their relationships with the personality traits of nurses	C	*N* = 669. 82.79% were women. The average age of participants was 27.22 (SD = 5.13) Age range was divided into three groups: equal or less than 25 years (45.27%), 26–30 years old (26.9%) and equal or older than 31 years (27.81%)	‐ Nomophobia Scale (Yildirim and Correia, 2015) ‐ Big Five‐Factor Personality Traits Inventory (Sumer et al., 2005)	Nurses who used mobile phones more during the day showed higher levels of nomophobia. In addition, a relationship was observed between the level of nomophobia and neuroticism scores	Risk factors ⟶ nomophobia: Age (≤ 25 vs. 26–30 vs. ≥ 31) ⟶ nomophobia: F = 9.521^∗^ (−); post hoc: ≤ 25 > 26–30, ≥ 31. Gender (female vs. male) ⟶ nomophobia: t = 3.516∗ (+). Professional experience (≤ 1 y vs. 2–5 y vs. ≥ 6 y) ⟶ nomophobia: F = 4.266^∗^ (−); post hoc: ≤ 1 y, 2–5 y > ≥ 6 y. Institution experience (≤ 1 y vs. 2–5 y vs. ≥ 6 y) ⟶ nomophobia: F = 3.700^∗^ (−); post hoc: ≤ 1 y, 2–5 y > ≥ 6 y. Daily smartphone use time (1–2 h vs. 3–4 h vs. ≥ 5 h) ⟶ nomophobia: F = 17.782^∗^ (+); post hoc: ≥ 5 h > 3–4 h > 1–2 h. Daily internet use time (1–2 h vs. 3–4 h vs. ≥ 5 h) ⟶ nomophobia: F = 16.284^∗^ (+); post hoc: ≥ 5 h > 3–4h > 1–2 h. Checking frequency per hour (≤ 5 vs. ≥ 6 times/hour) ⟶ nomophobia: t = −4.840^∗^ (+). Carrying a charger (yes vs. no) ⟶ nomophobia: t = 3.989^∗^ (+). Spending time on the smartphone before sleep (yes vs. no) ⟶ nomophobia: t = 6.922^∗^ (+). Bedtime smartphone shutdown (yes vs. no) ⟶ nomophobia: z = 2.679 (+). Checking the smartphone immediately after waking (yes vs. no) ⟶ nomophobia: t = 7.774^∗^ (+) Thinking of yourself as a smartphone addict (yes vs. no) ⟶ nomophobia: t = 10.956^∗^ (+).	80% (4/5) Moderate–high quality

Vitale et al. [33], Italy	To investigate which risk factors are strongly associated with nomophobia in Italian nurses, according to sociodemographic characteristics, physical activity habits, body mass index, anxiety and depression	C	*N* = 430. 75.58% were women. The average age of participants was 43.16 (SD = 11.44) Age range was from under 30 years old to 61 years old, with a predominance of participants between 31 and 40 years old (40.7%)	‐ Hospital Anxiety and Depression Scale (Zigmond and Snaith, 1983). ‐ Nomophobia Questionnaire (Yildirim and Correia, 2015)	Nomophobia affected more women, and its prevalence decreased with age. Nurses with low levels of physical activity or high levels of anxiety reported higher levels of nomophobia. Nurses with low to moderate levels of nomophobia did not suffer from depression. There was no statistically significant difference between nomophobia and body mass index	Risk factors ⟶ nomophobia (group comparisons)e Sex (female vs. male) ⟶ nomophobia level: *χ* ^2^, *p* < 0.001^∗∗∗^ (+)e. Age group ⟶ nomophobia level: *χ* ^2^, *p* < 0.001^∗∗∗^ (−)e. Years of work experience ⟶ nomophobia level: *χ* ^2^, *p* < 0.001^∗∗∗^ (−)e. Shift work ⟶ nomophobia level: *χ* ^2^, nse. Nursing educational level ⟶ nomophobia level: *χ* ^2^, (ns)e. Body mass index category ⟶ nomophobia level: *χ* ^2^, nse. Nomophobia ⟶ mental and physical health: Anxiety ⟶ nomophobia: *β* = 0.237^∗∗∗^ (+); t = 4.277; 95% CI: [0.083, 0.224]; VIF = 1.533. Physical activity ⟶ nomophobia: *β* = −0.221^∗∗∗^ (−); *t* = −4.773; 95% CI: [−0.153, −0.064]; VIF = 1.071. Depression ⟶ nomophobia: β = 0.072, ns; *t* = 1.294; *p* = 0.196; VIF = 1.527. Body mass index ⟶ nomophobia: *β* = −0.009 (ns); *t* = −0.197 (ns); VIF = 1.054. Correlations (rs) ↔ nomophobia: Anxiety ↔ nomophobia: rs = 0.302^∗∗∗^ (+). Depression ↔ nomophobia: rs = 0.231^∗∗∗^ (+). Physical activity ↔ nomophobia: rs = −0.241^∗∗∗^ (−). Body mass index ↔ nomophobia: rs = 0.108, ns	100% (5/5) High quality

Xue et al. [34], China	To explore the present status of work procrastination among clinical nurses and to identify possible profile categories. Furthermore, to analyze the impact of cell phone addiction and demographic determinants on work procrastination among nurses	C	*N* = 1.418. 95.28% were women. The average age of participants was 34.13 (SD = 9.75) Age range: 20–59 years old	‐ Procrastination at work scale (Wang et al., 2021) ‐ Mobile phone addiction index (Leung, 2008)	The mean work procrastination score of nurses was 21.00. In addition, mobile phone addiction and work environment were influential factors in the mean procrastination score of nurses	Smartphone addiction ⟶ Clinical practice (work procrastination)f: Smartphone addiction (total score): C1 = 33.00 (26.00–39.00) vs. C2 = 42.00 (33.50–49.00) vs. C3 = 51.00 (51.00–51.00); *Z* = 393.866^∗^ (+). Inability to control craving: C1 = 11.00 (8.00–13.00) vs. C2 = 14.00 (11.00–17.00) vs. C3 = 21.00 (21.00–21.00); *Z* = 443.887^∗^ (+). Feeling anxious and lost: C1 = 12.00 (9.00–16.00) vs. C2 = 15.00 (11.00–19.00) vs. C3 = 15.00 (15.00–15.00); Z = 96.970^∗^ (+). Withdrawal or escape: C1 = 6.00 (4.00–8.00) vs. C2 = 8.00 (6.00–9.00) vs. C3 = 9.00 (9.00–9.00); *Z* = 206.194^∗^ (+). Productivity loss: C1 = 3.00 (2.00–4.00) vs. C2 = 5.00 (4.00–6.00) vs. C3 = 6.00 (6.00–6.00); *Z* = 434.717^∗^ (+). Multivariate logistic regression (work procrastination profiles): C2 vs C1: smartphone addiction ⟶ C2 membership: β = 0.079^∗^ (+); OR = 1.082 (95% CI: 1.066–1.099) Model R2: NR. C3 vs. C1: smartphone addiction ⟶ C3 membership: *β* = 0.186^∗^ (+); OR = 1.205 (95% CI: 1.176–1.235) Model R2: NR	100% (5/5) High quality

Yang et al. [35], China	To explore the impact of nomophobic behaviors on the perceived clinical decision‐making of nurses in hospitals	C	*N* = 272. 86.8% were women. The average age of participants was 31.53 (SD = 6.29). Age range: 22–46 years old	‐ Clinical Decision‐Making in Nursing Scale (Jenkins, 1985) ‐ Nomophobia Questionnaire (Ren et al., 2020)	A moderate level of nomophobia was found in nurses. Furthermore, as nomophobia levels increased, the perception of clinical decision‐making decreased	Risk factors ⟶ nomophobia: Gender (female vs. male) ⟶ nomophobia: *t* = −2.152^∗^ (+). Age groups ⟶ nomophobia: *F* = 5.663^∗∗^ (−). Education level ⟶ nomophobia: *F* = 19.636^∗∗∗^ (direction varies by group; overall group differences). Duration of smartphone ownership ⟶ nomophobia: *F* = 5.040^∗∗^ (+). Daily smartphone usage duration ⟶ nomophobia: *F* = 14.089^∗∗∗^ (+). Nomophobia ⟶ Clinical practice (decision‐making perceptions) Nomophobia ⟶ clinical decision‐making perceptions: *r* = −0.365^∗∗∗^ (−). Regression model: *β* = −0.365^∗∗∗^ (−). *F* = 41.466^∗∗∗^. *B* = −0.304^∗^. Model *R*2 = 0.133 (13.3% variance explained). Nomophobia dimensions ⟶ clinical decision‐making perceptions (Pearson): Fear of being unable to obtain information ⟶ decision‐making: *r* = −0.418^∗∗∗^ (−). Fear of losing convenience ⟶ decision‐making: *r* = −0.362^∗∗∗^ (−). Fear of losing contact ⟶ decision‐making: *r* = −0.261^∗∗∗^ (−). Fear of losing Internet connection ⟶ decision‐making: *r* = −0.345^∗∗∗^ (−)	100% (5/5) High quality

Bautista and Lin [36], Philippines	To identify sociotechnical interactions by analyzing users, policy, and technology that affect nurses’ use of personal cell phones at work	QS	*N* = 23. 57% were women. The average age of participants was 26.57. Age range: 23–45 years old	Semistructured in‐depth interviews were conducted.	The sociotechnical analysis indicated that nurses used their mobile phones in various ways because they facilitated their work, despite policies prohibiting them. In addition, they inevitably disrupted their routines, but the nurses justified it for work purposes, as some hospitals did not provide mobile phones at work	NR (qualitative study; no quantitative effect estimates)	100% (5/5) High quality

*Note:* β, standardized regression coefficient; B, unstandardized regression coefficient; CI, confidence interval; F, one‐way ANOVA; R2, coefficient of determination; r, Pearson correlation coefficient; rs, Spearman correlation coefficient; t, independent samples *t*‐test; *χ* ^2^, chi‐square test; z, Mann–Whitney U test; (+) = positive association; (−) = negative associationaDescriptive data only: no statistical association tests performed;bGroup comparisons only (p‐values reported; test statistics such as t/F/*χ* ^2^ not reported in the article);cns (non‐significant path/indirect effect as reported);dChi‐square association reported with p value (*χ* ^2^ test); *χ* ^2^ statistic not reported;eGroup comparisons/descriptive only (*χ* ^2^ across categories; no effect coefficient reported);fBonferroni correction applied (as reported). C, cross‐sectional study; C1, low procrastination; C2, mid‐low procrastination; C3, mid‐high procrastination; D, design; FoMO, fear of missing out; USA, the United States; y, years. PMDs, portable/mobile devices; PMDs use, portable/mobile device use.

Abbreviations: DS = daytime sleepiness; ESS = Epworth Sleepiness Scale; MMAT = Mixed Methods appraisal tool; NE = nursing errors; NEQ = Nursing Error Questionnaire; NR = not reported; ns = not significant; OCF = occupational cognitive failure; OCFQ = Occupational Cognitive Failure Questionnaire; OR = odds ratio; PSQI = Pittsburgh Sleep Quality Index; QS = qualitative study; SD = standard deviation; SQ = sleep quality; VIF = variance inflation factor.

^∗^ *p* < 0.05.

^∗∗^ *p* < 0.01.

^∗∗∗^ *p* < 0.001.

The data synthesis revealed four categories related to mobile phone use: use in the clinical setting and associated risk factors, effects on mental and physical health, consequences on nurses’ clinical practice, and strategies. These four themes are presented narratively in the following and further discussed to provide broader evidence in the Discussion section.

### 3.3. Problematic Mobile Phone Use Outcome and Strategies Used

As previously mentioned, problematic mobile phone use is also referred to as mobile phone addiction, excessive mobile phone use, mobile phone dependence, or nomophobia. The term “problematic mobile phone use” has been uniformly applied throughout this review to describe the negative consequences of such use among nurses and the strategies to address these issues.

#### 3.3.1. Mobile Phone Use and Risk Factors

Studies indicated that nurses used their mobile phones for 2–5 h daily [22, 25, 30–32, 35]. In addition, one study found that nurses checked their phones an average of five times per hour during their shifts [35], while another study reported that 34.9% of nurses observed colleagues using their phones between 2 and 5 times per shift [24]. With respect to the most common personal activities performed using mobile phones during clinical shifts, six studies highlighted that the primary activity among nurses was the use of social media [23–25, 27, 35, 36]. Moreover, four studies identified other personal activities performed by nurses during their clinical shifts using mobile phones, such as sending text messages or making phone calls [23–25, 35]. The studies included in this review showed that most nurses had problematic mobile phone use. Specifically, three studies reported a level of mobile phone addiction between 39.45 (range: 0–85) and 66.50 (range: 0–104) among nurses [18, 26, 29]. In addition, seven studies found an average nomophobia level score between 50.80 and 78.17 (range: 20–140) among nurses [22, 25, 27, 28, 31, 32, 35]. To address the magnitude of associations requested, effect estimates and statistical test results reported by the primary studies are summarized in Table 2. Daily smartphone use showed a positive association with nomophobia (*β* = 0.355, *p* < 0.001), whereas age showed an inverse association (*β* = −0.115, *p* < 0.05) [22]. Similarly, five studies found that problematic phone use among nurses increased with longer daily mobile phone use [22, 28, 31, 32, 35]. Consistently, daily smartphone use duration was positively correlated with nomophobia in emergency nurses (*r* = 0.253, *p* < 0.001) [28], while nomophobia decreased with age (*r* = −0.238, *p* = 0.001) and work experience (*r* = −0.254, *p* = 0.001) [27] (Table 2). In addition, two studies found that nurses with more problematic mobile phone use increased their frequency of use during the workday by between 10% and 27% [22, 35].

In relation to risk factors, the gender of participants was found to be associated with problematic mobile phone use. Specifically, four studies reported higher levels of problematic use among female nurses compared to their male counterparts [28, 32, 33, 35]. However, gender differences were not statistically significant in some samples (e.g., *p* > 0.05) [28] (Table 2). In addition, six studies indicated that age influenced problematic mobile phone use [22, 24, 27, 30, 32, 35]. Specifically, three studies found that younger nurses reported higher levels of problematic use [24, 27, 30], while four studies observed a decrease in problematic use with increasing age among nurses [22, 32, 35]. These patterns were supported by significant group differences (e.g., age groups: *F* = 5.663, *p* < 0.01) [35] and by negative associations (Table 2). Regarding years of work experience, five studies found that nurses with less than ten years of work experience reported higher levels of problematic mobile phone use [24, 25, 27, 32, 33]. Work experience showed an inverse correlation with nomophobia (*r* = −0.254, *p* = 0.001) [27], and group comparisons suggested higher nomophobia among less experienced nurses (*F* = 3.047, *p* = 0.032) [25] (Table 2). With regard to academic background, one study found that nurses with bachelor’s degree showed greater problematic mobile phone use [35], while three studies found no statistically significant differences between academic background and problematic mobile phone use [25, 28, 33].

#### 3.3.2. Consequences on Mental and Physical Health

In nine studies included in this systematic review, problematic mobile phone use was found to have negative effects on nurses’ health, affecting both their mental health [18, 22, 24, 25, 27, 29, 30, 32, 33] and physical health [25, 30, 33]. The effect estimates and statistical test results are summarized in Table 2.

Concerning mental health, problematic mobile phone use among nurses was associated with anxiety in three studies [18, 29, 33] and with emotional exhaustion, depersonalization, cynicism, and burnout syndrome in two studies [18, 29]. Problematic mobile phones were positively associated with burnout (*r* = 0.33, *p* < 0.01) [18]. In addition, regression models indicated that problematic mobile phone use explained 18.9% of the variance in emotional exhaustion (*R* ^2^ = 0.189) and 25.8% of the variance in cynicism (*R* ^2^ = 0.258) [29] (Table 2). One study also reported a correlation with perceived workload and the fear of missing out (FoMO) in social settings [27]. Specifically, problematic mobile phone use was correlated with FoMO (*r* = 0.558, *p* < 0.01) [27], and a regression model explained 6% of the variance in perceived work overload (Model *R* ^2^ = 0.062; nomophobia *β* = 0.267) [27] (Table 2).

With regard to physical health, problematic mobile phone use among nurses was associated with sedentary behavior [33]. In addition, two studies linked this type of use to lower sleep quality, increased daytime sleepiness, and fatigue [25, 30]. In the study examining presleep mobile phone use was associated with poorer sleep quality (*β* = 0.38, *p* < 0.001), and the model explained 14.5% of sleep quality variance (*R* ^2^ = 0.145) and 30.5% of daytime sleepiness variance (*R* ^2^ = 0.305) [30] (Table 2).

#### 3.3.3. Consequences in Clinical Practice

In clinical practice, four studies associated problematic mobile phone use among nurses with distraction [23, 24, 26, 36]. The quantitative effect estimates and statistical test results are summarized in Table 2. In three studies, this problematic use showed a strong positive correlation with both active and passive workplace procrastination [26, 29, 34]. The problematic mobile phone use correlated positively with active procrastination (*r* = 0.533, *p* < 0.001) and passive procrastination (*r* = 0.468, *p* < 0.001) [26]. Regression models indicated that problematic mobile phone use explained 25% of the variance in active procrastination (model *R* ^2^ = 0.319; *β* = 0.516) and 18% of the variance in passive procrastination (model *R* ^2^ = 0.270; *β* = 0.437) [26] (Table 2). Moreover, four studies reported that nurses with higher levels of problematic mobile phone use experienced reduced attention and mindfulness, which negatively affected their self‐efficacy and clinical performance [23–25, 31]. In one study, nomophobia showed negative correlations with mindfulness (*r* = −0.289, *p* < 0.01) and self‐efficacy (*r* = −0.278, *p* < 0.01) [31] (Table 2). In two studies, increased problematic use was associated with a decline in perceived ability to make clinical decisions [28, 35]. In particular, problematic mobile phone use was negatively associated with clinical decision‐making perceptions (*r* = −0.365, *p* < 0.001), and the regression model explained 13.3% of the variance (model *R* ^2^ = 0.133; *β* = −0.365) [35]. In emergency nurses, problematic mobile phone use explained 53% of the variance in decision‐making perceptions (model *R* ^2^ = 0.530; *β* = −0.730) [28] (Table 2). This problematic use of mobile phones was found in six studies to have negative consequences on patient safety, quality of care, and the attention provided by nurses [18, 23, 24, 36]. Specifically, four studies reported a strong positive correlation between problematic mobile phone use and an increase in medication errors [18, 23, 24, 30]. In one study, higher addiction scores were observed among nurses reporting adverse events (e.g., wrong medication: *t* = −3.62, *p* < 0.001; pressure ulcer: *t* = −3.09, *p* = 0.002; falls with injury: *t* = −3.06, *p* = 0.002; unplanned extubation: *t* = −3.58, *p* < 0.001) [18] (Table 2). Regarding quality of care, one study found an increase in the incidence of pressure ulcers, patient falls, unplanned extubations, and healthcare‐associated infections in units where nurses exhibited higher levels of problematic mobile phone use [18]. Furthermore, a study identified that problematic mobile phone use in a clinical setting led to a deterioration in communication with patients and a loss of patient confidentiality and privacy when phones were used to document results or make digital copies of patient records, which were sometimes shared with other colleagues [36].

#### 3.3.4. Strategies

Regarding strategies to prevent and address problematic mobile phone use in the clinical setting, six studies highlighted the need to include continuing education courses on self‐management of mobile phone use that enable nurses to use these devices responsibly during working hours and in their personal lives [22, 26, 28, 31, 35, 36]. On the other hand, two studies emphasized the importance of incorporating content related to mobile phone use in clinical settings into undergraduate nursing education [33, 35]. Similarly, three studies demonstrated that implementing psychological interventions can reduce stress and anxiety among nurses exhibiting problematic mobile phone use [26, 31, 35]. On the other hand, six studies highlighted that nursing managers are crucial in the development and implementation of strategies based on the early identification of addictive behaviors toward these devices through periodic assessments by nurses, as well as policies that regulate mobile phone use in the clinical environment by establishing criteria for the appropriate use of these devices to ensure patient safety and preserve the health of nurses [18, 23, 25, 31, 35, 36]. Finally, one study suggested that the provision of mobile phones by the hospital during working hours, with access to applications and online resources that support clinical work, could alleviate problematic mobile phone use in the clinical setting by limiting the use of personal mobile phones during working hours [36].

### 3.4. Quality Assessment of Studies Included

Generally, of the 16 studies assessed, their methodological quality was considered to be good; eleven studies were rated as 100% quality criteria met, three studies were rated as 80% criteria met, while the remaining two studies were rated as 60% criteria met. More specifically, among them, the only one qualitative study has all ratings of “Yes” in terms of the appropriate approach to answer the research question, appropriate data collection procedure, sufficient results interpretation, and coherence between data source and analysis [36]. Besides, five quantitative descriptive studies have all ratings of “Yes” in terms of appropriate sampling strategy, appropriate sample representative of the target population, right measurement, low nonresponse bias, and appropriate statistical analysis [18, 22, 25, 28, 35]. Also, five quantitative cross‐sectional analytic studies have all ratings of “Yes” in terms of an appropriate sample representative of the target population, right measurement, complete outcome data, and all accounted confounders accounted for in the design and analysis and the intervention administered as intended [26, 29, 31, 33, 34]. Concerning the weakness of the included studies, two quantitative nonrandomized studies have one rating of “No” in terms of the first criterion as an appropriate sample representative of the target population [30], and in terms of the fourth criterion, confounding factors were not taken into account in the design [32]. Regarding quantitative descriptive studies, two have one rating of “Can not tell” in terms of the fourth criterion as not reporting nonresponse bias and not reporting reasons for nonresponse [24, 27]. One quantitative descriptive study has one rating of “No” in terms of the second criterion, as the measurements were appropriate, did not provide information on the validity, and only provided reliability values [23]. One quantitative descriptive study has one rating of “No” in terms of the fifth criterion as the dyadic statistical analyses used were not clearly stated and justified [23]. The participants in one quantitative descriptive study did not state clear descriptions of the target population and thus rated the second question as “No” [27]. The detailed information for quality evaluation is outlined in Table 3.

**TABLE 3:** Quality assessment of the included studies according to MMAT.

Qualitative studies	Is the qualitative approach appropriate to answer the research question?	Are the qualitative data collection methods adequate to address the research question?	Are the findings adequately derived from the data?	Is the interpretation of results sufficiently substantiated by data?	Is there coherence between qualitative data sources, collection, analysis, and interpretation?	Comments^a^ (%)
Bautista and Lin [36]	Yes	Yes	Yes	Yes	Yes	100 (5/5)

**Quantitative nonrandomized studies (cross-sectional analytic studies)**	**Are the participants representative of the target population?**	**Are measurements appropriate regarding both the outcome and intervention (or exposure)?**	**Are there complete outcome data?**	**Are the confounders accounted for in the design and analysis?**	**During the study period, is the intervention administered (or exposure occurred) as intended?**	Comments**^a^** (%)

El‐Sayed et al. [26]	Yes	Yes	Yes	Yes	Yes	100 (5/5)
Ma et al. [18]	Yes	Yes	Yes	Yes	Yes	100 (5/5)
Pormehr et al. [30]	No	Yes	Yes	Yes	Yes	80 (4/5)
Sharma et al. [31]	Yes	Yes	Yes	Yes	Yes	100 (5/5)
Uguz and Bacaksiz [32]	Yes	Yes	Yes	No	Yes	80 (4/5)
Vitale et al. [33]	Yes	Yes	Yes	Yes	Yes	100 (5/5)
Xue et al. [34]	Yes	Yes	Yes	Yes	Yes	100 (5/5)

**Quantitative descriptive studies**	**Is the sampling strategy relevant to address the research question?**	**Is the sample representative of the target population?**	**Are the measurements appropriate?**	**Is the risk of nonresponse bias low?**	**Is the statistical analysis appropriate to answer the research question?**	Comments**^a^** (%)

Bülbüloğlu et al. [22]	Yes	Yes	Yes	Yes	Yes	100 (5/5)
Conant et al. [23]	Yes	Yes	No	Yes	No	60 (3/5)
Di Simone et al. [24]	Yes	Yes	Yes	Can not tell	Yes	80 (4/5)
Dinçer and Çelik Ínce [25]	Yes	Yes	Yes	Yes	Yes	100 (5/5)
Hoşgör et al. [27]	Yes	No	Yes	Can not tell	Yes	60 (3/5)
Kapikiran et al. [28]	Yes	Yes	Yes	Yes	Yes	100 (5/5)
Ma et al. [29]	Yes	Yes	Yes	Yes	Yes	100 (5/5)
Yang et al. [35]	Yes	Yes	Yes	Yes	Yes	100 (5/5)

^a^Quality criteria met. 5, 100% quality criteria met; 4, 80% quality criteria met; 3, 60% quality criteria met.

## 4. Discussion

This study provides a synthesis of risk factors, negative outcomes, and potential strategies related to problematic mobile phone use amongst nurses, highlighting its implications for health and clinical efficiency.

With regard to problematic mobile phone use, it was observed to be a highly prevalent behavior amongst nurses, with levels of nomophobia ranging from moderate to severe across the majority of studies analyzed. These findings confirm that problematic mobile phone use is an emerging public health issue [13], which underscores the need for healthcare institutions and policymakers to develop and implement specific interventions to improve both the health of nurses and patient care outcomes [26]. Nurses with problematic mobile phone use were those who most frequently used these devices during clinical shifts, with the primary activity being social networking site (SNS) use and exhibiting compulsive checking behaviors. In this regard, previous studies report that the most predominant mobile phone addiction behaviors are compulsive checking of calls or messages and excessive concern about missing information posted on social networks [37, 38]. It is important to highlight that mobile phone use in the clinical setting may offer potential benefits for clinical communication and access to information [6], but its inclusion as a clinical resource may have facilitated the development of dependency amongst nurses [39]. Similarly, nurses working in units with high workloads report using their mobile phones for personal purposes during working hours as a coping mechanism for stress, emotional fatigue, or work overload, resorting to social networks as a means of escapism and immediate gratification [25, 27, 36]. This maladaptive coping mechanism is consistent with gratification theory, whereby individuals resort to potentially addictive behaviors to temporarily alleviate negative emotional states [36]. However, this short‐term relief may ultimately exacerbate stress levels amongst nurses by contributing to task accumulation and reduced job satisfaction [25, 27]. These findings support the notion that nursing is frequently considered a stressful profession, which may increase the risk of developing problematic mobile phone use as an avoidance strategy [40]. Likewise, our findings demonstrated that the age, work experience, and gender of nurses may be risk factors in the development of problematic mobile phone use. Younger nurses, particularly those with less experience, may exhibit behavioral patterns related to mobile phone use that could hinder their professional development and patient care [24, 25, 27, 30, 32, 33]. This trend could be attributed to their profound familiarity with mobile phones, which has generated new challenges in the clinical setting that require awareness‐raising and training initiatives on appropriate mobile phone use that should be implemented in nursing programs and clinical settings. Furthermore, younger nurses demonstrated lower self‐regulation skills in relation to mobile phone use in clinical settings [24]. In this regard, self‐regulation capabilities and control over mobile phone use tend to improve with age [37]. Similarly, more experienced nurses may have developed a stronger professional identity and internalized professional norms that prioritize patient care over personal device use [29]. Regarding gender, female nurses showed a higher level of problematic mobile phone use compared to male nurses. Nevertheless, it should be emphasized that this finding must be interpreted with caution, given that the nursing workforce is predominantly female (between 32.6% and 100% women across the different studies), which may limit the generalization of gender comparisons. These identified risk factors may contribute to establishing profiles of nurses who are more vulnerable to developing problematic mobile phone use, thereby facilitating early identification and the development of personalized interventions aimed at preventing problematic use of these devices. Future research should explore whether specific combinations of risk factors (e.g., youth, high workload, and low resilience) generate synergistic vulnerability, which would enable risk stratification and the design of targeted prevention programs.

Our findings indicate that problematic mobile phone use has negative consequences on the mental and physical health of nurses. Likewise, problematic mobile phone use can be considered a significant predictor of perceived work overload and burnout amongst nurses, explaining up to 25.8% of the variance in cynicism and 18.9% of the variance in emotional exhaustion [18, 27, 29]. These effect sizes indicate that problematic mobile phone use is not only associated with burnout but may play a relevant causal role in its development [18]. Similarly, problematic mobile phone use was associated with neurotic personality traits, increasing the tendency to experience negative emotions and behavioral disorders [32]. The identification of neuroticism as a vulnerability factor suggests that personality‐based assessment could improve risk detection [41]. Furthermore, the results report that individuals with problematic mobile phone use demonstrate difficulties in regulating negative emotions, thus adopting maladaptive coping strategies [17, 42]. In this context, resilience is considered a protective factor in the development of addictive behaviors and contributes to preserving mental health [43]. Therefore, nurse managers could implement strategies aimed at strengthening resilience and encouraging nurses to adopt positive coping mechanisms to prevent the development of problematic mobile phone use and promote mental well‐being. On the other hand, problematic mobile phone use was related to a sedentary lifestyle, decreased sleep quality, increased daytime sleepiness, and fatigue. A recent study concludes that sleep disturbances are caused by prolonged exposure to mobile phone screens, negatively affecting the mental well‐being of nurses [30]. Reducing mobile phone use in the clinical setting shows progressive improvement in the psychological well‐being of nurses with problematic mobile phone use by reducing cognitive overload and promoting emotional self‐regulation [22, 25]. These findings emphasize the need to address this addictive behavior and regulate mobile phone use in the clinical setting.

Another relevant finding concerned the consequences in the clinical setting of problematic mobile phone use amongst nurses. In this regard, mobile phone use in the clinical setting causes frequent interruptions that hinder task resumption, increasing the risk of clinical errors and reducing response capacity in complex care contexts [8, 44]. Moreover, the data obtained revealed that problematic mobile phone use amongst nurses negatively affects clinical safety and the quality of care provided to patients. In this sense, problematic mobile phone use explained 25% of the variance in active procrastination and 18% in passive procrastination and, more significantly, up to 53% of the variance in perceptions of clinical decision‐making amongst emergency nurses, indicating a reduction in their essential professional competences [26, 27]. Similarly, interruptions caused by mobile phones divert attention from communication toward the device, resulting in decreased eye contact, active listening, and empathy, essential aspects for identifying and responding to patient needs [8]. Ineffective communication with patients complicates the therapeutic relationship and the adequate exchange of information and may undermine patient trust, negatively affecting the quality of care [45]. Furthermore, Ma et al. [18] highlight the increase in nosocomial infections in units where nurses present higher levels of problematic mobile phone use [18]. Mobile phones may be contaminated with pathogens that could be transmitted to patients through hands; however, most hospitals have not implemented specific cleaning routines for these devices [46]. The potential for mobile phones to harbor pathogens poses serious risks for infection control, which highlights the need to improve policies in clinical settings. Likewise, it was identified that inappropriate mobile phone use in the clinical setting may compromise patient confidentiality and privacy [36]. Beyond legal implications, these practices undermine the therapeutic trust relationship between patients and nurses, which is essential for ensuring quality care [47]. These findings underscore the importance of supervising the work of nurses in order to improve the quality of care provided to patients. In this regard, supervision of nursing tasks reduces care omissions and improves the efficiency of nurses [48]. Similarly, nursing managers should implement policies that promote ethical awareness regarding the appropriate use of these devices during patient care.

Regarding strategies to prevent and address problematic mobile phone use amongst nurses reported in the selected literature, the need for specific training on the responsible use of these devices during working hours and awareness‐raising about problematic mobile phone use to prevent addictive behaviors and promote appropriate use of these devices in both personal and work settings is highlighted [22, 26, 28, 31, 35, 36]. Such training should extend beyond simple awareness‐raising to include skill development components, such as techniques for managing FoMO, strategies for establishing boundaries between device use at work and in the personal sphere, and mindfulness‐based approaches to reduce compulsive checking [17]. Education and awareness‐raising regarding the use of these devices are fundamental, as the effectiveness of interventions will be limited if nurses do not recognize that mobile phone use reduces the time dedicated to patient care [44]. Furthermore, guidelines on appropriate mobile phone use should be incorporated into university curricula so that future nurses develop healthy and responsible behaviors with these devices, ensuring patient safety and professional ethics [33, 35]. On the other hand, the development of psychological interventions in the clinical setting aimed at fostering resilience and adaptive coping behaviors, which can act as protective factors against problematic mobile phone use [26, 31, 35]. Similarly, psychological interventions aimed at reducing stress and anxiety amongst nurses could be effective in decreasing problematic mobile phone use, improving mental well‐being, attention, and concentration, and thereby work performance [26, 31, 35]. From an organizational perspective, nurse managers must implement institutional strategies and policies that enable early identification of these behaviors and regulate this practice, establishing periodic assessments of nurses and regulations on mobile phone use in the clinical setting [18, 23, 25, 31, 35, 36]. Such policies must strike a balance between restriction and autonomy, recognizing that nurses require a certain degree of connectivity to meet personal needs as well as for potential clinical uses [7]. Furthermore, Bautista and Lin [36] suggested that providing mobile phones by the hospital during working hours, with access to resources that support clinical work, could reduce the use of these devices for personal purposes.

## 5. Limitations

The results derived from this review should be considered in light of certain limitations. First, a systematic and rigorous research methodology was used, but it is possible that not all studies available in the literature were included. Furthermore, problematic mobile phone use is a recent phenomenon, and studies on nurses began to be published less than ten years ago. In this regard, more studies on the phenomenon are needed in order to identify and investigate the risk factors that influence its development, the consequences at the personal and clinical levels related to patient care, work dynamics and quality of care, as well as the development of strategies to prevent and address problematic mobile phone use. Likewise, the lack of consensus regarding terminology and measurement of problematic mobile phone use, with studies employing constructs of nomophobia, mobile phone addiction, or problematic use assessed via diverse instruments, complicates comparison across studies and synthesis of findings. Efforts toward standardized definitions and validated, cross‐culturally appropriate assessment tools would advance the field. Finally, publication bias may influence the literature, with studies reporting null or counterintuitive findings less likely to be published, potentially inflating the apparent strength of associations identified in this review. Similarly, the scarcity of previous studies, the heterogeneity of the results and measures used to assess the phenomenon, and its consequences and strategies made it difficult to synthesize the results and carry out a meta‐analysis.

## 6. Conclusion

The synthesis of the literature on problematic mobile phone use among younger nurses shows that it is a widespread phenomenon that negatively affects their mental and physical health and clinical performance. Specifically, at the mental level, an increase in anxiety, stress, emotional exhaustion, depersonalization, cynicism, perceived work overload, and burnout was found. At the physical level, nurses with more problematic mobile phone use show less physical activity and sleep disturbances. The negative consequences described in their professional work negatively affect safety, quality of care, and patient care. Nursing managers need to develop and implement strategies to prevent and mitigate problematic mobile phone use among nurses and raise awareness about the appropriate use of these devices in the clinical setting.

## 7. Implications for Practice

This systematic review focused on an emerging phenomenon such as problematic mobile phone use among nurses to highlight the negative effects it can have on nurses’ health and clinical work. Nurses are essential to ensuring optimal patient care, and the high prevalence of this phenomenon could negatively affect the quality of care provided. This review allows us to identify the consequences of problematic use on their health and care work, establishing a starting point for developing and implementing effective strategies to mitigate the consequences of problematic mobile phone use. Therefore, nursing managers should establish effective institutional policies that regulate the use of mobile phones by nurses during their workload. In addition, nursing managers could regularly assess nurses’ levels of mobile phone addiction or nomophobia, which would enable early identification and intervention. It would also be necessary to develop training courses on the proper use of these devices in order to prevent the development of addictive behaviors and promote responsible use during working hours. Similarly, the implementation of clear recommendations on the disinfection and handling of mobile phones could reduce the risk of nosocomial infections in vulnerable patients. In addition, protocols must be established regarding the appropriate use of mobile phones in the clinical setting to ensure clinical safety, confidentiality, and ethics in patient care and to increase the clinical performance of nurses. On the other hand, developing strategies and interventions to reduce excessive mobile phone use can increase mental well‐being, reduce professional burnout, and improve your health and clinical work. Based on the above, it is necessary to develop experimental studies to evaluate the effectiveness of interventions to prevent and address the development of this phenomenon.

## Author Contributions

Gema López‐Gutiérrez was involved in conceptualization, methodology, investigation, data curation, and writing−original draft. Vanesa Gutiérrez‐Puertas was involved in conceptualization, methodology, investigation, writing−original draft, writing−review and editing, validation, and project administration. Blanca Gómez‐Guerrero was involved in methodology, investigation, data curation, and writing−original draft. Hélder Jaime Fernandes was involved in methodology, investigation, data curation, and writing−original draft. Stefanos Mantzoukas was involved in conceptualization, methodology, investigation, and writing−review and editing. Lorena Gutiérrez‐Puertas was involved in conceptualization, methodology, investigation, resources, writing−original draft, writing−review and editing, validation, project administration, and supervision.

## Funding

This study was supported by the PPIT 2024‐2025‐University of Almeria’s programme for research and knowledge transfer (2024‐2025).

## Conflicts of Interest

The authors declare no conflicts of interest.

## Data Availability

The data that support the findings of this study are available from the corresponding author upon reasonable request.
